# Epstein–Barr virus-associated retinitis: a case report

**DOI:** 10.3389/fmed.2026.1835638

**Published:** 2026-07-24

**Authors:** Weiwei Wang, Libin Zheng, Qinxiang Zheng, Liguo Feng

**Affiliations:** 1 Department of Nursing, Sir Run Run Shaw Hospital, Zhejiang University School of Medicine, Hangzhou, China; 2 Department of Ophthalmology, Sir Run Run Shaw Hospital, Zhejiang University School of Medicine, Hangzhou, China

**Keywords:** case report, Epstein–Barr virus, polymerase chain reaction, retinitis, vitritis

## Abstract

**Purpose:**

To report the clinical features, diagnostic workup, and management of Epstein–Barr virus (EBV)-associated retinitis in a patient without overt clinical evidence of severe immunodeficiency.

**Case description:**

A 33-year-old Chinese man presented with a 1-month history of blurred vision, redness, and photophobia in the left eye. Ocular examination revealed vitritis, white snowball-like vitreous deposits, optic disc edema, yellow-white retinal lesions, and tortuous retinal veins. Optical coherence tomography and fluorescein angiography supported active posterior segment inflammation. Initial vitreous smear and bacterial and fungal cultures were negative. Polymerase chain reaction testing of a vitreous sample detected EBV DNA at 123 copies/mL, whereas herpes simplex virus (HSV), varicella-zoster virus (VZV), and cytomegalovirus (CMV) were negative. The patient received systemic acyclovir combined with corticosteroid therapy. Intraocular inflammation improved markedly, but pars plana vitrectomy was later performed because of persistent organized vitreous opacity and retinal traction. At the 4-month follow-up, retinal lesions had largely resolved and best-corrected visual acuity improved to 0.2.

**Conclusion:**

This case suggests that EBV should be considered in the differential diagnosis of atypical infectious retinitis. Vitreous PCR may provide important etiological evidence, and timely combined medical and surgical management may contribute to a favorable outcome.

## Introduction

Epstein–Barr virus (EBV) is a ubiquitous human herpesvirus that establishes lifelong latency after primary infection (1, 2). Although EBV infection is common in the general population, ocular involvement is uncommon, and EBV-associated retinitis has been reported only sporadically (3–5). Posterior segment manifestations include retinitis, retinal vasculitis, papillitis, and, in some cases, acute retinal necrosis (6). Because these presentations are rare and may overlap with other infectious or non-infectious entities, diagnosis can be challenging in clinical practice (7). The diagnosis of infectious retinitis increasingly relies on microbiological analysis of intraocular fluids, particularly polymerase chain reaction (PCR), which can provide important etiological evidence and help guide timely therapeutic decisions (7–9). This is especially relevant in atypical cases in which fundus findings may mimic fungal endophthalmitis, syphilis, lymphoma, or other viral retinitides (10). In addition, the optimal management of EBV-associated retinitis remains incompletely defined because of the limited number of reported cases and the heterogeneity of treatment strategies (11).

Here, we report a case of EBV-associated retinitis with vitritis and papillitis in a patient without overt clinical evidence of severe immunodeficiency. The diagnosis was supported by vitreous PCR, and the patient showed anatomical improvement after systemic antiviral and corticosteroid therapy followed by pars plana vitrectomy. This case highlights the diagnostic value of vitreous PCR and the importance of timely individualized management in atypical posterior segment inflammation.

## Case presentation

A 33-year-old Chinese man presented with a 1-month history of blurred vision, redness, and photophobia in the left eye. Before referral to our hospital, he had been diagnosed elsewhere with macular edema and branch retinal vein occlusion and had received oral prednisone for approximately 2 weeks; however, the exact dose and regimen could not be confirmed from the available records. The rationale for the prior diagnosis and corticosteroid treatment could not be fully verified because the detailed ophthalmic records from the referring institution were unavailable. His medical history was notable for renal calculi for 3 months and hepatitis B virus infection for 10 years. Before the onset of ocular symptoms, he had undergone two nephrolithotomy procedures and developed fever after the second operation, for which he received intravenous levofloxacin and piperacillin/tazobactam for 10 days.

**FIGURE 1:**
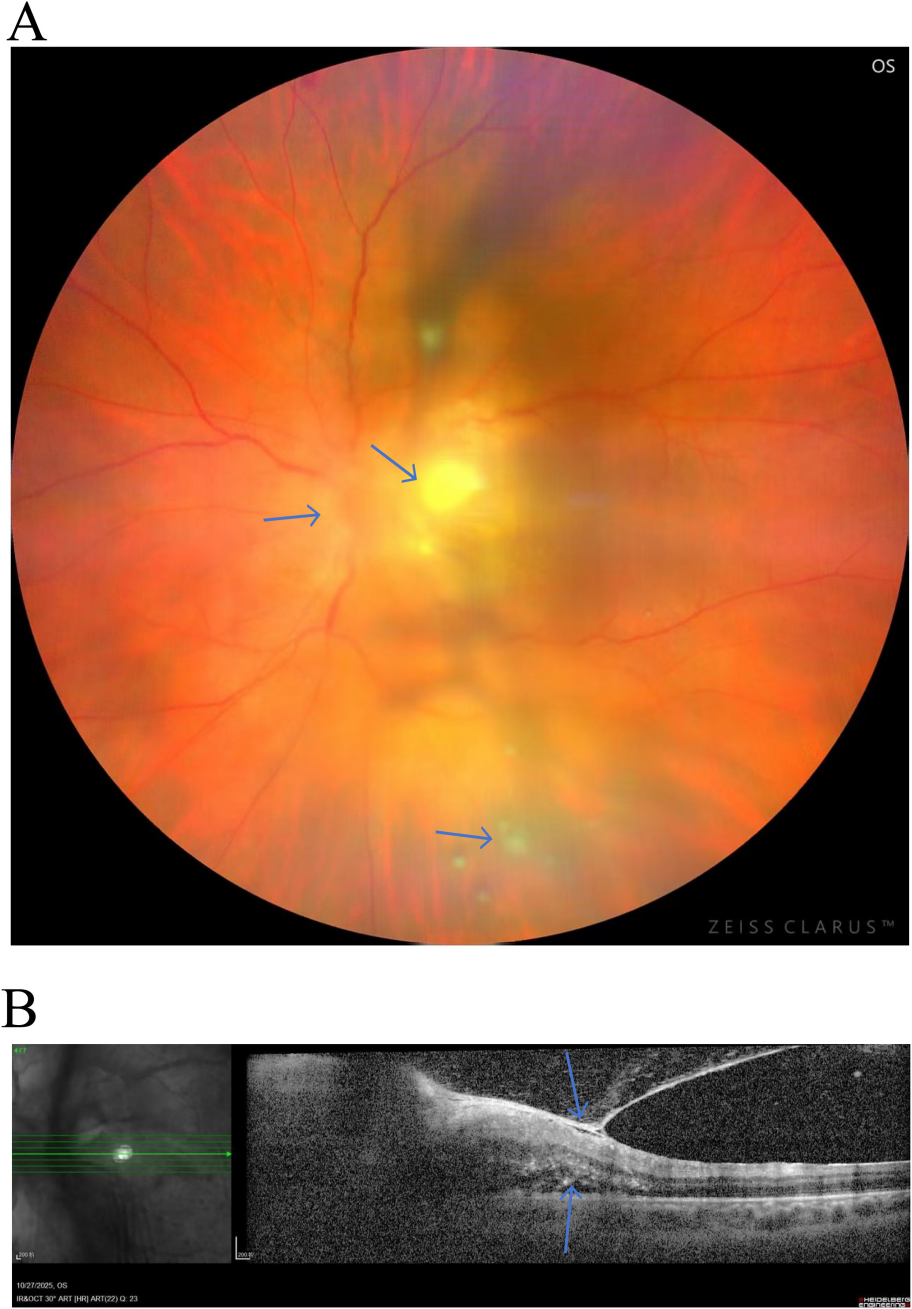
Ocular findings at presentation in the left eye. **(A)** Color fundus photography showed marked vitreous opacity with white snowball-like vitreous deposits, optic disc edema, yellow-white lesions over the posterior retinal surface, and tortuous retinal veins. **(B)** Optical coherence tomography showed an organized posterior vitreous cortex with adhesion to the retinal surface, associated with vitreoretinal traction and scattered hyperreflective retinal spots. Blue arrows indicate the main lesions.

**FIGURE 2:**
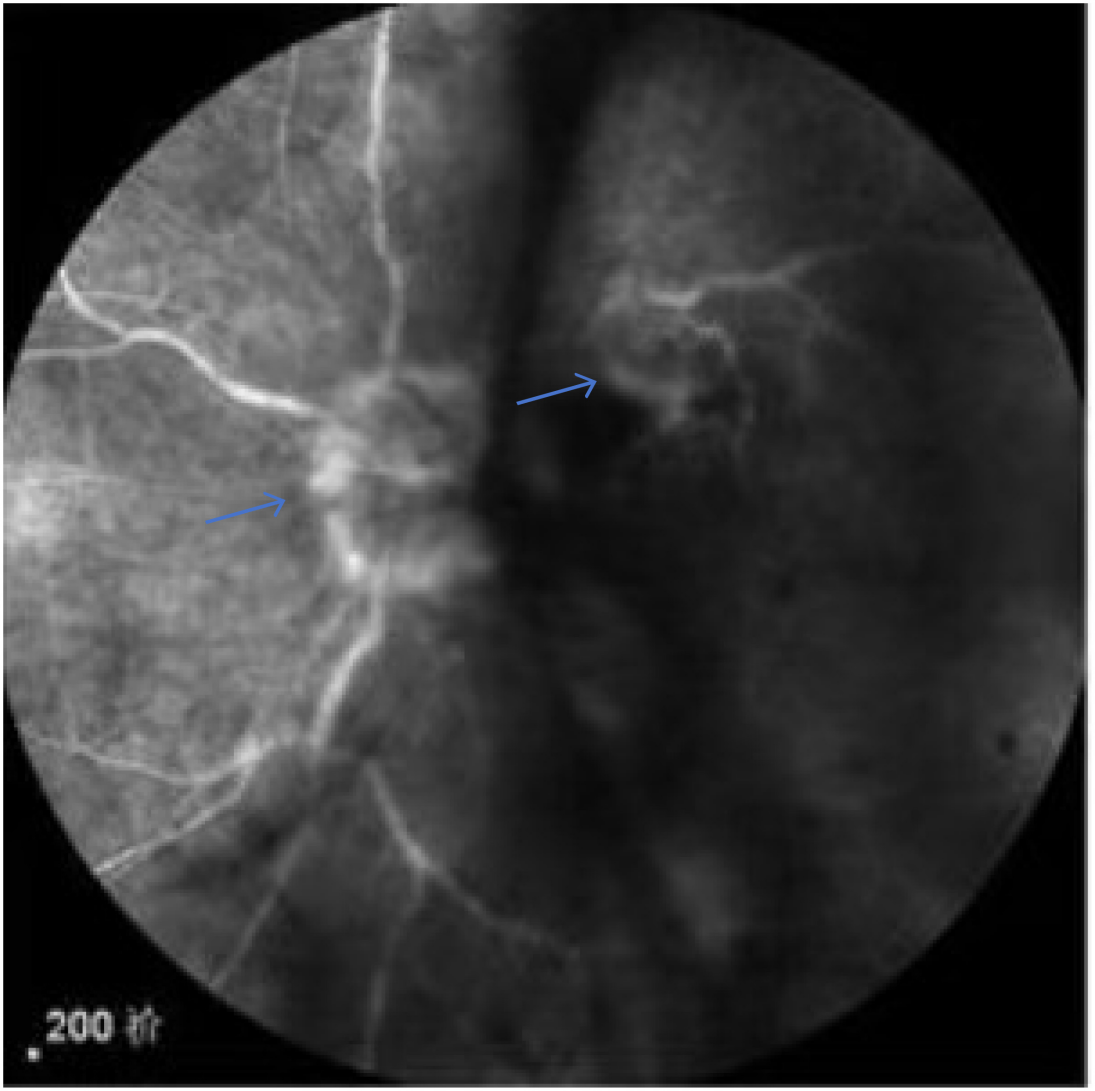
Fluorescein angiography at presentation in the left eye. Fluorescein angiography showed optic disc hyperfluorescence and patchy hyperfluorescence of the posterior retinal lesions, indicating active inflammatory involvement of the posterior segment. Blue arrows indicate the main lesions.

On presentation, the best-corrected visual acuity was finger counting at 60 cm in the left eye and 1.0 in the right eye. Intraocular pressure was 15.0 mmHg in the left eye and 18.3 mmHg in the right eye. The right eye was unremarkable. Slit-lamp examination of the left eye showed mild conjunctival congestion, 0.5 + cells in the anterior chamber, and 3 + cells in the anterior vitreous. Fundus examination revealed marked posterior vitreous opacity, white snowball-like vitreous deposits, optic disc edema, yellow-white lesions over the posterior retinal surface, and tortuous retinal veins (Figure 1A). Optical coherence tomography showed an organized posterior vitreous cortex and retinal thickening with multiple hyperreflective spots (Figure 1B). Fluorescein angiography demonstrated early hyperfluorescence of the optic disc and retinal lesions, with increased hyperfluorescence in the late phase, indicating active posterior segment inflammation (Figure 2).

Laboratory testing showed a white blood cell count of 12,300 cells/μL (reference range, 3,500–9,500 cells/μL), a C-reactive protein level of 0.6 mg/L (reference range, <6.0 mg/L), and a procalcitonin level of 0.04 ng/mL (reference range, <0.05 ng/mL). Serum EBV IgM was 3.9 U/ml (reference range, <20 U/ml). Serum HSV and CMV antibodies were also undetectable. Other infectious and inflammatory factors, including syphilis, toxoplasmosis, tuberculosis, HIV-related immunosuppression were negative. Based on the ocular findings, fungal endophthalmitis was initially suspected. An intravitreal injection of 5 μg amphotericin B was administered immediately after admission, and a vitreous sample was obtained for smear as well as bacterial and fungal cultures. However, intraocular inflammation became more severe on the following day. Smear results were negative, and no bacterial or fungal growth was observed on culture after 1 week. Antifungal treatment was therefore discontinued.

**FIGURE 3:**
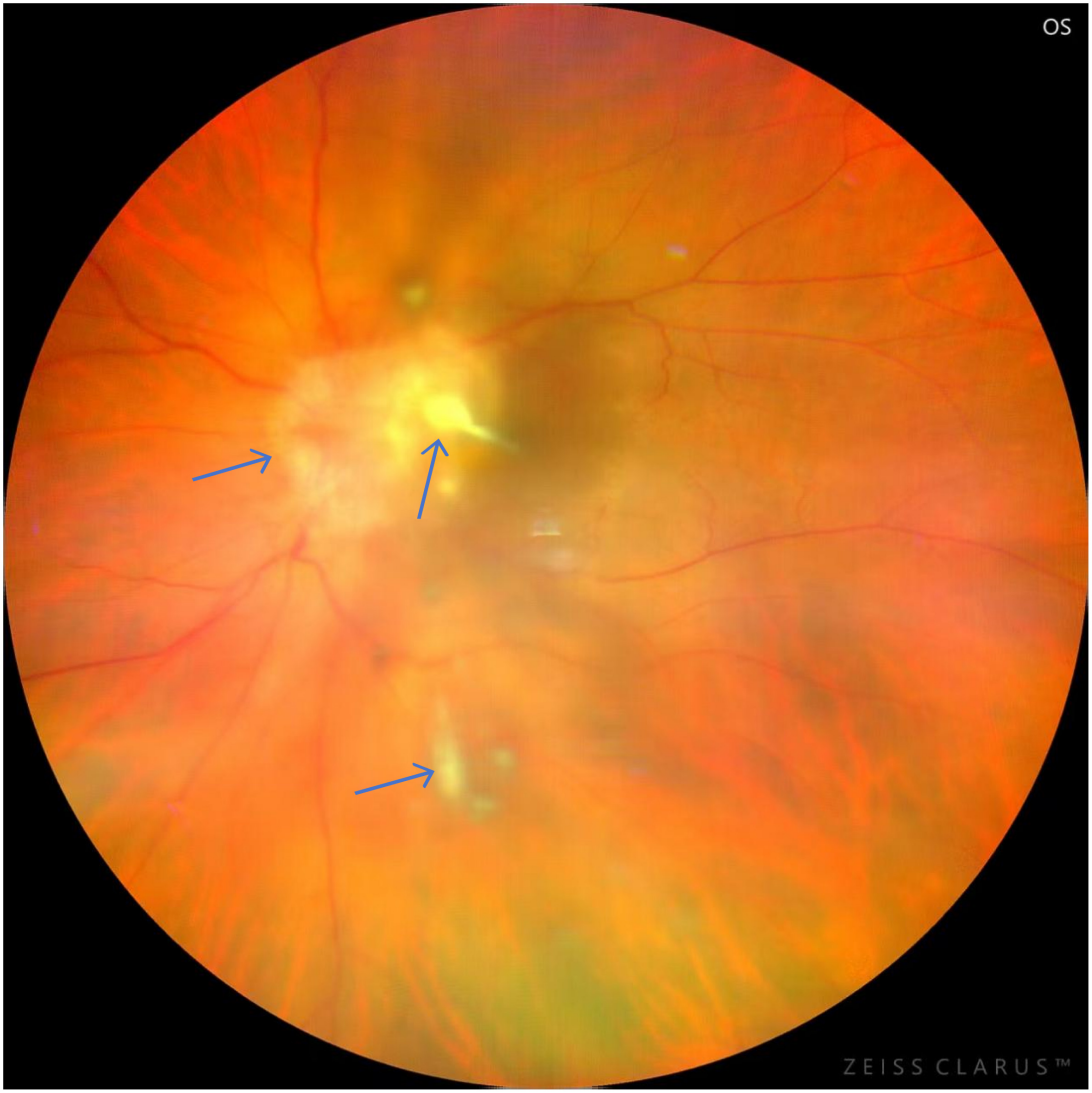
Fundus findings after 2 weeks of treatment in the left eye. Color fundus photography showed marked improvement in vitreous opacity, with a reduction in white snowball-like vitreous deposits, shrinkage of the yellow-white retinal lesions, decreased retinal venous tortuosity, and partial resolution of optic disc edema. Blue arrows indicate the main lesions.

A second vitreous biopsy was then obtained for quantitative real-time polymerase chain reaction (PCR) and inflammatory factors. At the same time, the patient was started empirically on intravenous acyclovir (500 mg every 8 h for 5 days) and methylprednisolone (40 mg/day), followed by oral acyclovir (400 mg, 5 times daily). PCR of the vitreous sample detected EBV DNA at 123 copies/mL, whereas herpes simplex virus (HSV), varicella-zoster virus (VZV), and cytomegalovirus (CMV) were negative. The ratio of IL-10/IL-6 was 0.0044. To confirm the result, real-time PCR was repeated in our laboratory and again showed EBV positivity, with a cycle threshold value of 37.05, while the other tested herpesviruses remained negative. One day after treatment initiation, posterior segment inflammation began to subside. Five days later, repeat PCR of the vitreous sample was negative for EBV, HSV, VZV, and CMV. Also, Serum EBV-IgM was still negative (3.5 U/ml). After 2 weeks of treatment, the vitreous snowball-like deposits had markedly decreased, the yellow-white retinal lesions had shrunk, retinal venous tortuosity had improved, and optic disc edema had largely resolved (Figure 3).

**FIGURE 4:**
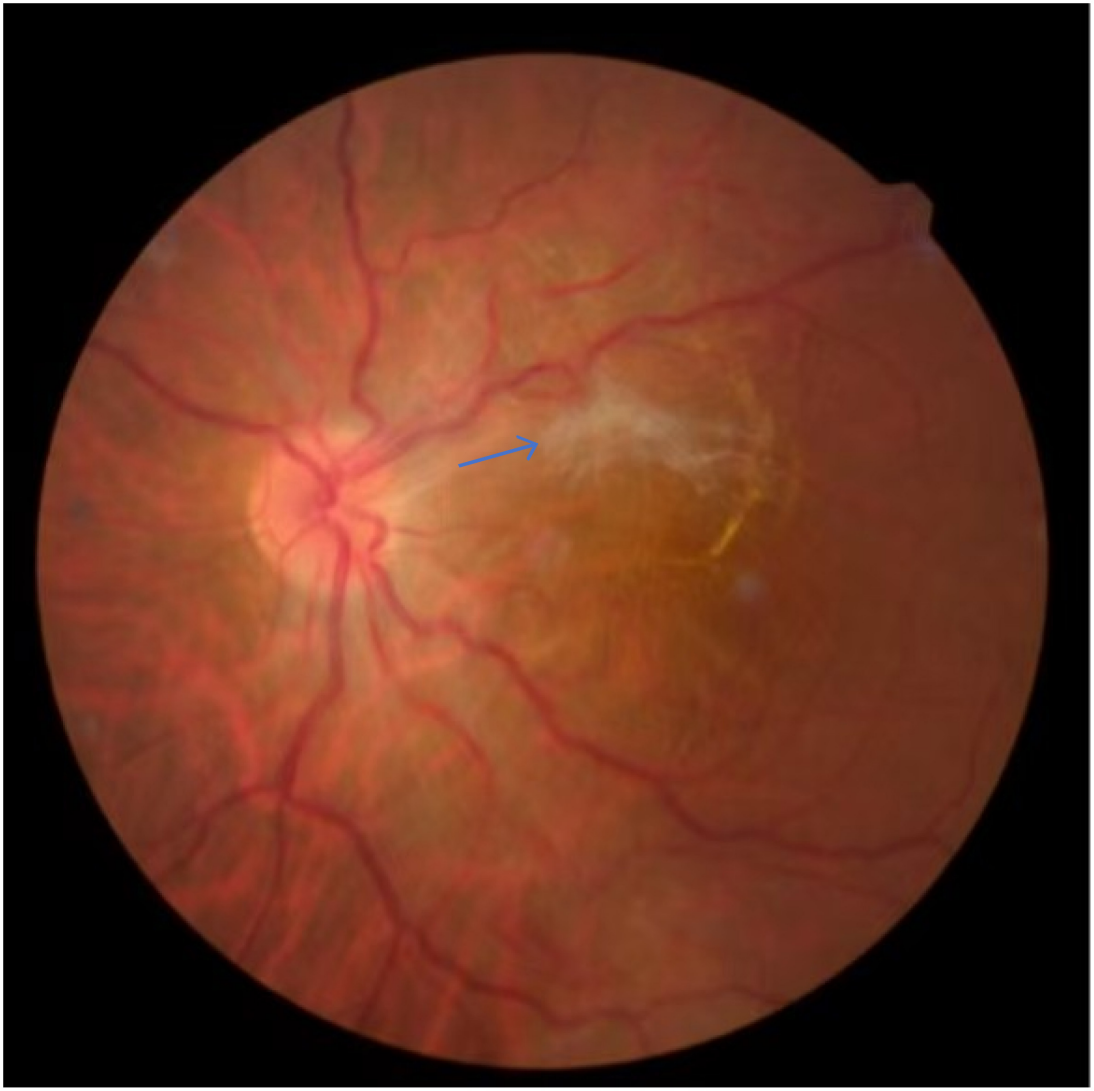
Fundus findings at 4-month follow-up in the left eye. Color fundus photography showed marked anatomical recovery, with near-complete resolution of the retinal lesions and residual macular pucker along the superotemporal vascular arcade. Blue arrows indicate the main lesions.

Because organized vitreous opacity and persistent traction on the retinal surface continued to limit visual function, 23-gauge pars plana vitrectomy was performed 3 weeks after the initiation of conservative treatment. PCR of the vitreous sample obtained at that time was again negative for viral DNA. At the 4-month follow-up visit, best-corrected visual acuity in the left eye had improved to 0.2. The retina was nearly normal, except for macular pucker along the inferotemporal vascular arcade (Figure 4).

Key clinical events, diagnostic testing, treatment, and outcomes are summarized in Table 1.

**TABLE 1:** Timeline of key clinical events, diagnostic testing, management, and outcomes.

Timepoint	Events and key findings
10 years prior	Hepatitis B virus infection (carrier status).
∼3 months prior	Renal calculi diagnosed.
Before ocular symptom onset	Two nephrolithotomy procedures; fever after the second procedure; IV levofloxacin + piperacillin/tazobactam for 10 days.
∼1 month prior	Left-eye blurred vision, redness, and photophobia began.
Pre-referral (∼2 weeks)	Outside diagnosis: macular edema/branch retinal vein occlusion; oral prednisone for ∼2 weeks (exact dose/regimen unavailable).
Presentation (Day 0)	BCVA (LE): finger counting at 60 cm; dense vitritis with snowball deposits, optic disc edema, yellow-white posterior retinal lesions; OCT/FA supported active posterior inflammation.
Day 0	Empirical intravitreal amphotericin B; vitreous sampling for smear and bacterial/fungal culture (negative).
Diagnostic confirmation	Vitreous qPCR: EBV DNA 123 copies/mL; HSV/VZV/CMV negative; systemic acyclovir + corticosteroids initiated.
∼2 weeks	Marked reduction of vitreous deposits and retinal lesions; improvement of optic disc edema/vascular tortuosity.
∼3 weeks	Pars plana vitrectomy for persistent organized vitreous opacity and vitreoretinal traction.
4 months	BCVA (LE) improved to 0.2; near-complete lesion resolution; residual macular pucker.

## Discussion

This case describes EBV-associated retinitis with vitritis and papillitis in a patient without overt clinical evidence of severe immunodeficiency. Although EBV infection is highly prevalent worldwide, EBV-associated posterior segment disease has been reported only sporadically. Previous reports have shown that EBV-related ocular involvement may present with different phenotypes, including punctate outer retinitis, retinal vasculitis, papillitis, retinitis, and acute retinal necrosis (ARN) (11). In this context, the present case adds to the limited literature by demonstrating a pattern characterized by dense vitritis, white snowball-like vitreous deposits, optic disc edema, yellow-white retinal lesions, and retinal venous tortuosity.

Comparison with previously reported cases is clinically informative. Raymond et al. described punctate outer retinitis during acute EBV infection (12), whereas Mushiga et al. reported EBV retinitis accompanied by papillitis (10), which more closely resembles the inflammatory pattern seen in our patient. In contrast, Chan et al. described EBV-associated ARN in an immunocompetent host, suggesting that EBV-related posterior segment disease may span a spectrum from non-necrotizing retinitis to fulminant necrotizing retinopathy (13). Our case appeared to fit better within the non-ARN inflammatory spectrum, because marked vitritis, papillitis, and retinal surface lesions were present, but the clinical course and retinal findings were not typical of classic necrotizing peripheral retinitis.

Another notable feature of the present case is that EBV was the only virus detected in the vitreous sample. This finding is relevant because published reports have suggested that EBV-associated retinitis or ARN is often identified in immunocompromised patients or in the setting of mixed viral infections, particularly together with VZV. In contrast, sole EBV positivity has been reported less frequently. Schaal et al. described sole EBV infection in a patient receiving immunosuppressive therapy with ARN, whereas our patient had no overt evidence of severe systemic immunodeficiency (14).

Nevertheless, his immune status should be interpreted cautiously because comprehensive immunological evaluation was not performed. In addition, chronic hepatitis B virus carriage, recent surgery, postoperative infection, prior systemic corticosteroid exposure, and prolonged antibiotic treatment may all have influenced host immune function. Therefore, rather than being definitively immunocompetent, the patient is more appropriately described as having no overt clinical evidence of severe immunodeficiency. This case further supports the possibility that isolated intraocular EBV infection may occur even outside the classic setting of profound immune compromise.

The interpretation of vitreous PCR findings also merits comparison with prior reports. Previous studies have shown that EBV may be detected in ocular fluid more often in immunocompromised patients and in selected complex uveitis cases, but the clinical significance of EBV positivity may be difficult to determine, particularly when the viral load is low (4, 15, 16). In our patient, the low-level positive result was considered clinically meaningful for several reasons. EBV was the only pathogen detected, the result was reproduced in a second assay performed in our laboratory, and EBV DNA became undetectable after treatment in parallel with clinical improvement. In addition, the patient had experienced ocular symptoms for more than 1 month before presentation, and prior studies of viral retinitis have suggested that viral load in intraocular fluids may vary according to the interval between symptom onset and sampling. Although these considerations do not eliminate all uncertainty, they strengthen the interpretation that the PCR result reflected true intraocular involvement rather than incidental detection alone.

A noteworthy discrepancy in this case was the detection of EBV DNA in the vitreous sample despite undetectable serum EBV IgM antibodies. Several factors may account for this finding. First, serological testing was performed more than 1 month after symptom onset; by this time, acute-phase IgM antibodies may have waned below detectable levels. Second, the eye is an immunologically privileged site, and EBV reactivation or local persistence within the intraocular compartment may occur without eliciting a significant systemic humoral response, a phenomenon previously described in other viral retinitides. Third, although our patient did not have overt severe immunodeficiency, his immune status was not entirely unperturbed—chronic hepatitis B carriage, recent surgical stress, postoperative antibiotic use, and antecedent corticosteroid exposure may have contributed to a blunted systemic antibody production. Importantly, serum EBV DNA PCR was not performed in this case, so we cannot comment on the presence or absence of systemic viremia. We acknowledge that the lack of EBV IgG testing is another limitation; measuring IgG and EBNA antibodies would have helped confirm prior infection, which is nearly universal in the adult population. Taken together, these considerations support the interpretation that the positive vitreous PCR reflected true intraocular EBV involvement rather than incidental detection, despite negative systemic serology.

Our case also contributes to the discussion of treatment strategies. The previously cited literature reflects substantial heterogeneity in management. Keorochana reported successful treatment of EBV-associated retinal vasculitis using acyclovir alone (3). Mushiga et al. described a more intensive approach using oral valaciclovir together with intraocular methotrexate and intravitreal foscarnet (10). In another reported case of EBV-associated acute retinal necrosis, pars plana vitrectomy with silicone oil tamponade was required because of retinal detachment and proliferative vitreoretinopathy (17). Compared with those cases, our patient showed marked improvement after systemic acyclovir combined with corticosteroid therapy, but PPV was subsequently required because persistent organized vitreous opacity and retinal traction continued to limit visual recovery. Taken together, these reports suggest that there is no single standard treatment for EBV-associated retinitis; instead, management should be individualized according to the inflammatory severity, retinal involvement, virological findings, and structural complications (18).

The differential diagnosis in this case was also important. The initial dense vitreous opacity and white vitreous deposits raised concern for fungal endophthalmitis, which prompted intravitreal amphotericin B injection and vitreous sampling. However, empirical intraocular antifungal therapy before pathogen confirmation may carry a risk of retinal toxicity. Smear and culture remained negative, and the clinical course after antifungal treatment did not support a fungal etiology. In addition, systemic corticosteroids were started together with antiviral therapy to control severe intraocular inflammation, but early corticosteroid use in the setting of incompletely characterized infectious retinitis may theoretically aggravate infection if adequate antimicrobial coverage is lacking. In the present case, these potential risks underscore the importance of close clinical monitoring, prompt microbiological evaluation, and timely adjustment of treatment once etiological evidence becomes available. In this setting, vitreous PCR provided crucial etiological evidence and helped redirect treatment (19). This experience is consistent with previous reports emphasizing that microbiological testing of ocular fluid can be especially valuable in atypical retinitis cases that overlap clinically with fungal infection, syphilis, lymphoma, or other viral retinitides (5, 6).

## Conclusion

In conclusion, we report a case of EBV-associated retinitis with vitritis and papillitis in patients without overt clinical evidence of severe immunodeficiency. This case highlights that EBV should be considered in the differential diagnosis of atypical posterior segment inflammation, particularly when the clinical presentation overlaps with other infectious entities. Vitreous PCR, including viral load assessment, provided important etiological evidence and was valuable for guiding diagnostic reassessment and treatment adjustment. Combined medical therapy and pars plana vitrectomy, when indicated for persistent vitreoretinal traction, were associated with anatomical improvement and partial visual recovery in this patient.

## Data Availability

The original contributions presented in this study are included in this article/supplementary material, further inquiries can be directed to the corresponding author.
